# USP18 promotes cell proliferation and suppressed apoptosis in cervical cancer cells via activating AKT signaling pathway

**DOI:** 10.1186/s12885-020-07241-1

**Published:** 2020-08-08

**Authors:** Wenjing Diao, Qisang Guo, Caiying Zhu, Yu Song, Hua Feng, Yuankui Cao, Ming Du, Huifen Chen

**Affiliations:** 1 Medical Center of Cervical Diseases, Obstetrics and Gynecology Hospital, Fudan University, Fangxie Road No. 419, Shanghai, 200011 P.R. China; 2 Department of Laboratory Medicine, Shanghai First Maternity and Infant Hospital, Tongji University School of Medicine, Changle Road No.536 , Jingan District, Shanghai, 200040 P.R. China

**Keywords:** Cervical cancer, USP18, AKT, Proliferation, Apoptosis

## Abstract

**Background:**

The deubiquitinating (DUB) enzyme ubiquitin-specific protease 18 (USP18), also known as UBP43, is an ubiquitin-specific protease linked to several human malignancies. However, USP18’s underlying function in human cervical cancer remains unclear. In the current study, we aimed to analyse the role of USP18 and its signalling pathways in cervical cancer.

**Methods:**

Quantitative real-time polymerase chain reaction (qRT-PCR) and immunohistochemical staining were performed to analyse USP18 levels in cervical cancer and matched to adjacent normal tissues. Moreover, RNA interference (RNAi) and lentiviral-mediated vector transfections were performed to silence and overexpress USP18, respectively, in cervical cancer cells. Further, Cell Counting Kit-8 (CCK-8) and Annexin V/PI staining assays were used to assess its biological function in cell proliferation and apoptosis, respectively. A xenograft model was used to examine USP18’s function in vivo.

**Results:**

The present findings demonstrated that USP18 was overexpressed in cervical cancer specimens and cell lines. Silencing USP18 in SiHa and Caski cervical cancer cell lines inhibited cell proliferation, induced apoptosis, and promoted cleaved caspase-3 expression. In contrast, USP18 overexpression showed the opposite effects in human HcerEpic cells. A Gene Set Enrichment Analysis revealed that USP18 was enriched in the PI3K/AKT signalling pathway in cervical cancer. Hence, the PI3K/AKT inhibitor LY294002 was used to determine the relationship between USP18 and AKT in cervical cancer cells. Importantly, LY294002 significantly abolished the effects of USP18 overexpression in cervical cancer cells. In vivo, USP18 silencing inhibited human cervical cancer cells’ tumorigenicity.

**Conclusions:**

The current study indicates that USP18 is an oncogenic gene in cervical cancer. Our findings not only deepened the understanding of USP18’s biological function in cervical cancer pathogenesis, but we also provided novel insight for cervical cancer therapy.

**Trial registration:**

Retrospectively registered.

## Background

The main cause of cervical cancer, the fourth most common malignancy in women worldwide, is human papillomavirus (HPV) infections [1]. Although traditional therapies (radiotherapy or radical surgery) for cervical cancer are widely available, the clinical outcomes remain unsatisfactory. More than one-third of cervical cancer patients experience recurrence, inevitably leading to death [2]. The prognoses of metastatic cervical cancer patients remain poor, and the overall survival is limited to only 10 months [3]. Hence, understanding the molecular pathogenesis of cervical cancer is an essential step for developing novel therapies.

The deubiquitinating (DUB) enzyme ubiquitin-specific protease 18 (USP18), also known as UBP43, is a ubiquitin-specific protease [4]. A previous report indicated that USP18 is increased in certain human tumours [5]. Silencing USP18 inhibits the growth of mammary tumours in vivo and promotes the apoptosis of breast cancer cells [6, 7]. Moreover, USP18 silencing significantly increases the apoptosis of glioblastoma cells [8]. Further, knocking down USP18 suppresses cell growth and induces apoptosis in acute promyelocytic leukaemia [9]. Furthermore, USP18 promotes breast cancer growth by enhancing the activity of the AKT/Skp2 pathway [10]. However, USP’s underlying role and signalling pathway in cervical cancer cells requires further investigation.

The present study’s purpose was to explore USP18’s function in cervical cancer cells. We silenced and overexpressed USP18 in cervical cancer cells using RNA interference (RNAi) and lentiviral-mediated vector transfections, respectively. Our analyses not only elucidated USP18’s role but also identified its potential signalling pathway in cervical cancer cells.

## Methods

### Human tissues

The Shanghai First Maternity and Infant Hospital, Tongji University School of Medicine, Shanghai, China, provided 30 pairs of human cervical cancer and matched adjacent para-cancerous tissues. All cervical cancer patients provided written informed consent. The human cervical cancer and the corresponding adjacent normal tissues (*n* = 30) were surgically resected from cervival cancer patients in Shanghai First Maternity and Infant Hospital, Tongji University School of Medicine, Shanghai, China. The corresponding adjacent para-cancerous tissues were obtained 5 cm beyond the boundary of the cancerous tissue, which were myometrium in essence. Each samples were divided into two groups, one was harvested and embedded in Tissue-Tek OCT compound (Sakura, Tokyo, Japan) within 10 min after resection from the patients and subsequently snap-frozen in liquid nitrogen before storage at − 80 °C, while another one was fixed in fresh 10% neutral-buffered formal in for 24 h at room temperature.

### Cells culture

All cell lines used in the present study were purchased from the cell bank of the Shanghai Biology Institute (Shanghai, China), including normal cervical epithelial cells HcerEpic and the human cervical cancer cell lines, including Hela, C-33A, Caski, and SiHa. Cells were cultured in RPMI-1640 medium (SH30809.01B, Hyclone, USA) containing 10% foetal bovine serum (16000–044, Gibco, USA) and 1% penicillin-streptomycin (P1400–100, Solarbio, China) and maintained in a 37 °C incubator with 5% CO_2_. The AKT inhibitor LY294002 (25 μmol/L; S1105, Selleck, USA) was dissolved in dimethyl sulfoxide (DMSO, D2650, Sigma, USA) and used to treat the cells.

### Overexpression and knockdown of USP18

Briefly, the full-length USP18 (NM_017414.4) coding region sequence (CDS) was inserted into the lentiviral-mediator vector (pLVX-Puro). Then, the recombinant vector was transfected into human Hela cells using Lipofectamine 2000, following the manufacturer’s protocols (Cat: 11668027, ThermoFisher, USA) (oeUSP18). A mock vector was transfected as corresponding control (oeNC).

For silencing, three small interference RNAs (siRNAs), targeting different regions of the human *USP18* gene, were synthesised [siUSP18–1 (347–365): CCTGCTGCCTTAACTCCTT; siUSP18–2 (1004–1022): GCCAGATCCTTCC AATGAA; and siUSP18–3 (1023–1041): GCGAGAGTCTTGTGATGCT] and then transfected into Caski and SiHa cells. A nonspecific scrambled siRNA was transfected as corresponding control siNC (5′-CAACATTGGACAGACCTG CTGCCTT-3′).

### Cell proliferation

Cell proliferation was determined by using the Cell Counting Kit-8 (CCK-8) (CP002, SAB, USA) following the manufacturer’s instructions. The OD450 value was quantified using a microplate reader (DNM-9602, Pulangxin, China). Three replications were analysed for each time point.

### Flow cytometry

Briefly, the cell (Hela, Caski, and SiHa) were stained using an Annexin V-fluorescein isothiocyanate (FITC) apoptosis detection kit (Beyotime, China) according to the manufacturer’s instructions, at 48 h after transfection. Then, the proportion of apoptotic cells were determined using flow cytometer (BD, USA). Three replicates were necessary for each samples.

### Real-time PCR

The total RNA from cell samples was extracted using the TRIzol Reagent (1596–026, Invitrogen, USA). Then, the cDNA synthesis kit (Fermentas, Canada) was used to reverse transcribe the RNA into complementary DNA (cDNA) according to the manufacturer’s instructions. GAPDH expression was functioned as internal reference and used to normalise gene expression. Gene expressions were determined using the 2^-ΔΔCt^ method [11]. Three biological replicates were included for each analysis. The primers that used in this research were listed as follows: USP18 F 5′ TCTGGAG GGCAGTATGAG 3′, USP18 R 5′ TGGTAGTTAGGATTTCCGTAG 3′; and GAPDH F 5′ GGATTGTCTGGCAGTAGCC 3′, GAPDH R 5’ATTGT GAAAGGCAGGGAG 3′.

### Western blot

Total protein was extracted using RIPA lysis buffer (JRDUN, Shanghai, China). A BCA protein assay kit (PICPI23223, Thermo Fisher, USA) was used to measure total protein concentrations. Equal amounts of proteins adjusted to 25 μg were separated by 10% SDS-PAGE and subsequently transferred onto PVDF nitrocellulose membranes (HATF00010, Millipore, USA) for 12 h. After that, the membranes were then probed with primary antibodies at 4 °C overnight, followed by the appropriate HRP-conjugated goat anti-rabbit IgG (A0208, Beyotime, China) at 37 °C for 60 min. Protein signals were detected using a chemiluminescence system (5200, Tanon, China). GAPDH served as an endogenous reference. The protein expression was quantified as Gene _grey value_/GAPDH _grey value_. Each analysis was performed in triplicate. The primary antibodies that used the current study were listed as follows: USP18 (AB168478, Abcam, UK), cleaved caspase-3 (AB32042, Abcam, UK), AKT (#4691, CST, Danvers, USA), p-AKT (#4060, CST, Danvers, USA), Ki-67 (ab92742, Abcam, UK), Cyclin D1 (ab16663, Abcam, UK), Cleaved PARP (ab32064, Abcam, UK), Bax (ab32503, Abcam, UK), β-catenin (ab32572, Abcam, UK) and GAPDH (#5174, CST, Danvers, USA). Primary antibodies were detected using HRP-conjugated anti-rabbit IgG (A0208, Beyotime, Shanghai, China) or anti-mouse IgG (A0216, Beyotime, Shanghai, China) secondary antibodies.

### Immunohistochemistry

This assay was performed according to a previous reference [12]. In brief, The tissue sections were fixed in methanol (4%) for 30 min. Then, endogenous peroxidase activity was blocked by incubating with H_2_O_2_ (3%) for 10 min. The tissue sections were then incubated with the USP18 primary antibody (ab115618, Abcam, UK) at room temperature for 1 h, followed by the HRP-labelled secondary antibody for 30 min. Then, the sections were stained with DAB and re-stained with haematoxylin for 3 min. An upright microscope (ECLIPSE Ni, NIKON, Japan) was utilised to obtain images, which were analysed using the microscope image analysis system (DS-Ri2, NIKON, Japan) at a magnification of 200 × .

### Gene set enrichment analysis (GSEA)

The data were used to generate an ordered list of all genes according to their correlation with USP18 expression, and then a predefined gene set was given an enrichment score and *P* value. GSEA was performed using The Cancer Genome Atlas (TCGA) cervical cancer dataset with GSEA version 2.0.

### Xenograft model

All in vivo experiments were performed according to the Institute’s guidelines for animal experiments and approved by the independent ethics committee of Shanghai First Maternity and Infant Hospital, Tongji University School of Medicine, Shanghai, China.

All animals were treated in accordance with the Institutional Animal Care and Use Committee. An equal number of siNC or siUSP18 transfected Caski cells (*n* = 5 × 10^6^) were injected subcutaneously into the right flank of 4–6-week-old nude mice (*n* = 5 for each group; Shanghai Laboratory Animal Company, China). The length and width of the tumours were determined every 3 days for 33 days after cell injections. The volumes of the tumours were calculated according to the formula as follows: length × (width2/2). Then, both siNC and siTRIM37-injected mice were sacrificed by cervical dislocation, and then the tumour tissues were surgically removed and fixed in 4% formalin for further analysis.

### TUNEL staining

TUNEL assays were performed with sections using an ApopTag kit (Intergene) according to the supplier’s instructions. Three replicates were analysed for each sample.

### Statistical analysis

Statistical analyses were performed using GraphPad Prism software Version 7.0 (CA, USA). All data were presented as the mean ± S.E.M from three independent experiments. Statistical significance was assessed using Student’s t-test and one-way analysis of variance. A *p* value < 0.05 was considered to indicate statistical significance.

## Results

### USP18 is upregulated in human cervical cancer tissues

**Fig. 1:**
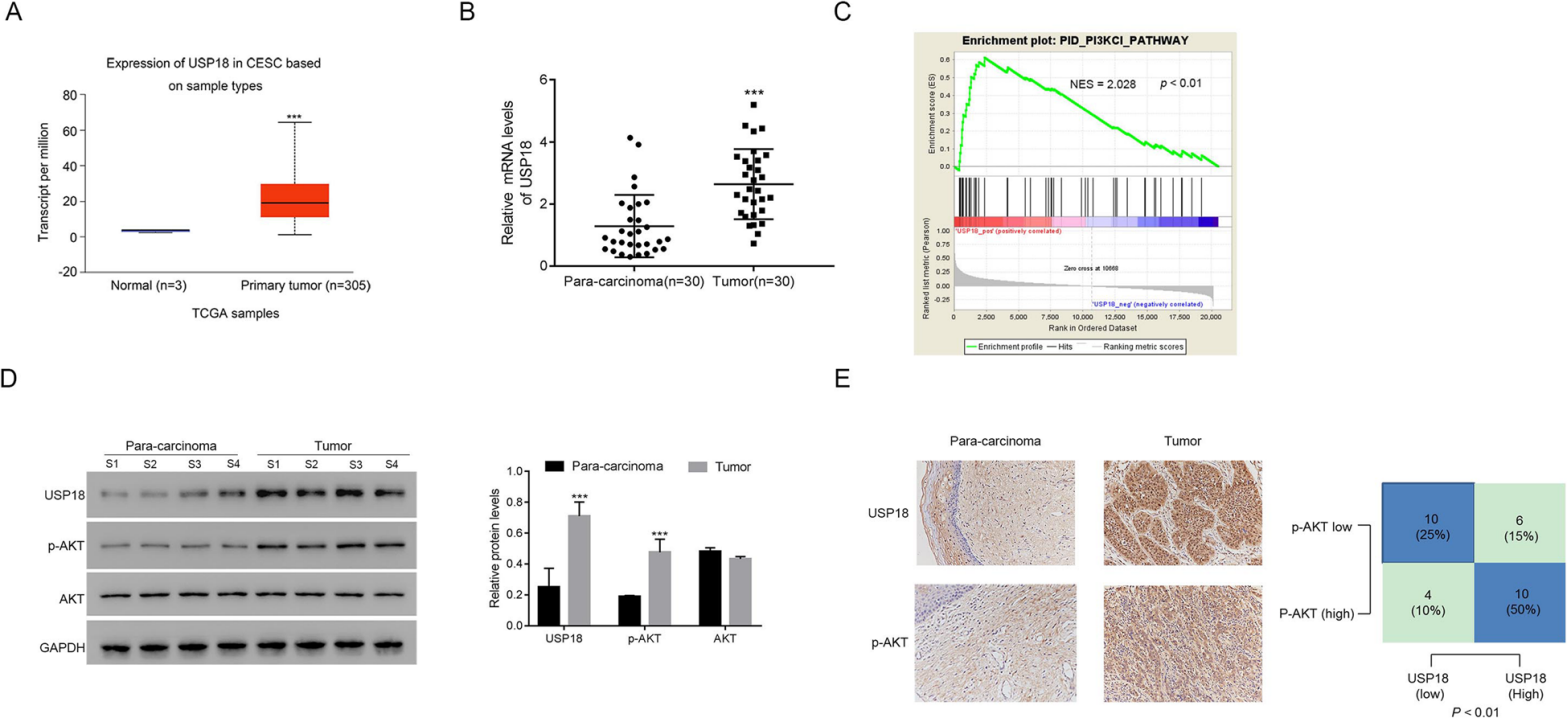
USP18 is upregulated in human cervical cancer tissues. **a** USP18 is upregulated in primary cervical cancer samples. Data collected from the TCGA database CESC dataset. *** *p* < 0.001 vs Normal. **b** The relative mRNA levels of USP18 were much higher in human cervical cancer tissues than that in para-cancer tissues, *n* = 30 for each group. *** *p* < 0.001 vs Normal. **c** USP18 was enriched in the PI3/AKT pathway. **d** The relative protein levels of USP18 and AKT phosphorylation were upregulated in human cervical cancer tissues compared with that in para-cancer tissues. *** *p* < 0.001 vs para-carcinoma tissues. *n* = 4 for each group. The full-length gels are presented in Supplementary Figure 1D. **e** IHC staining assay indicated the positive correlation between USP18 and AKT phosphorylation in human cervical cancer tissues, *n* = 30 for each group

To examine the relationship between USP18 and cervical cancer, we collected data from the UALCAN (http://ualcan.path.uab.edu/cgi-bin/TCGAExResultNew2.pl?genenam=USP18&ctype=CESC) database. As presented in Fig. 1a, the level of USP18 was substantially higher in cervical squamous cell carcinoma (CESC) samples than that in para-cancer samples. Additionally, 30 pairs of cervical cancer and matched adjacent para-cancer tissues were used to examine the mRNA level of USP18 further. Our results also indicated that USP18 was overexpressed in human cervical cancer tissues (Fig. 1b).

Moreover, GSEA indicated that USP18 was enriched in the PI3K/AKT signalling pathway (Fig. 1c). Next, Western blot analysis was used to examine the protein levels of USP18 and phosphorylated AKT (p-AKT) in four pairs of human cervical cancer and matched adjacent para-cancer tissues. As shown in Fig. 1d, both USP18 and p-AKT were upregulated significantly in human cervical cancer tissues. To further determine the relationship between USP18 and AKT, IHC staining assays were performed to determine the protein contents of USP18 and p-AKT in human cervical cancer and adjacent para-cancer tissues (*n* = 30). Correlation analysis indicated that USP18 was correlated with p-AKT expression in human cervical cancer tissues (Fig. 1e).

### Knockdown and overexpression of USP18 in human cervical cancer cells

**Fig. 2:**
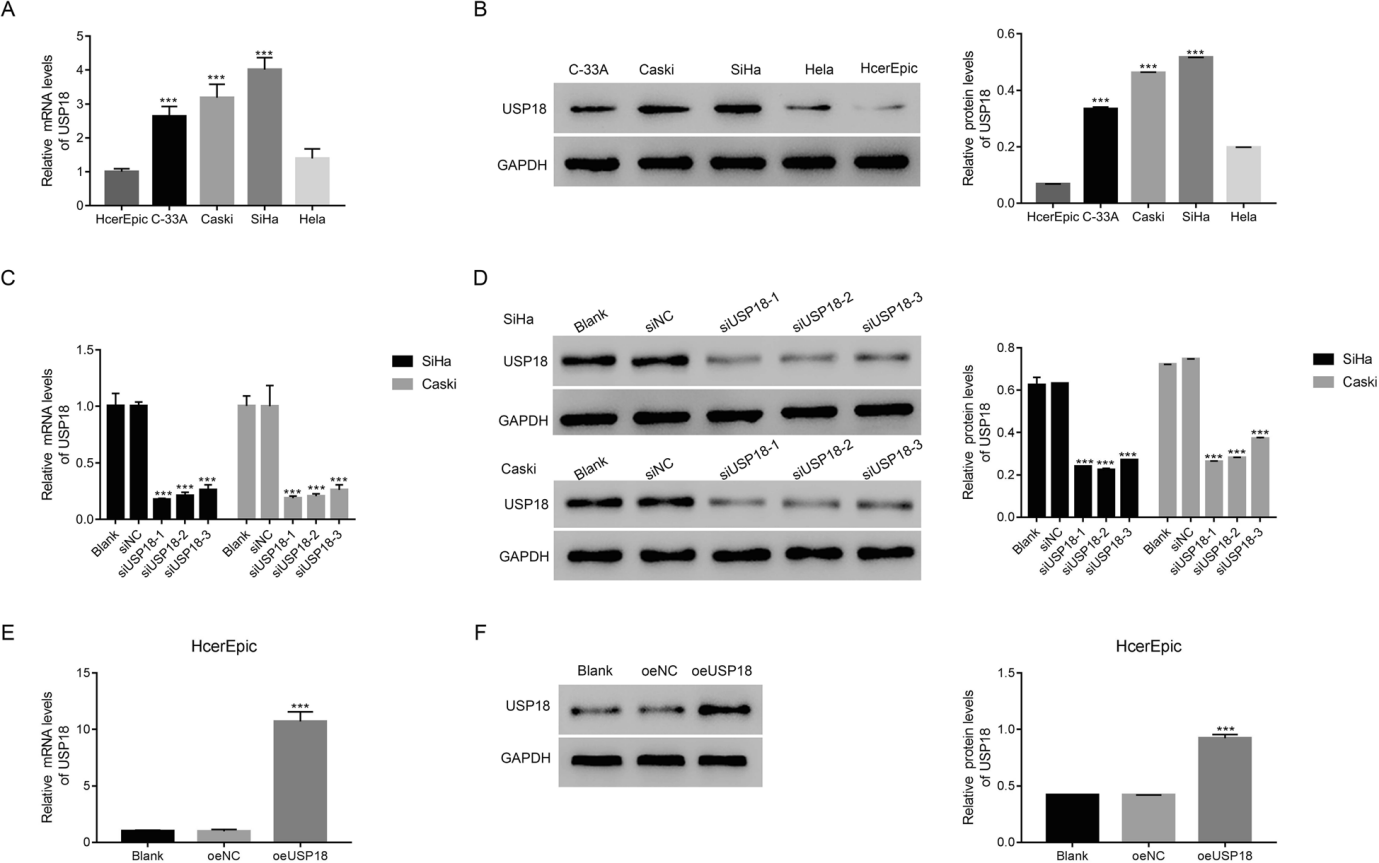
Knockdown and overexpression of USP18. **a** & **b** The relative mRNA and protein levels of USP18 were upregulated in C-33A, Caski, and SiHa cervical cancer cells, Three replications were analysed for each analysis. *** *p* < 0.001 vs HcerEpic. The full-length gels are presented in Supplementary Figure 2B. **c** & **d** USP18 siRNAs (siUSP18–1, siUSP18–2, and siUSP18–3) significantly suppressed the endogenous mRNA and protein levels of USP18 in SiHa and Caski cells. *** *p* < 0.001 vs siNC. Three replications were analysed for each analysis. The full-length gels are presented in Supplementary Figure 2D-up and Figure 2D-down. **e** & **f** oeUSP18 significantly improved the ectopic mRNA and protein levels of USP18 in HcerEpic cells. *** *p* < 0.001 vs oeNC. Three replications were analysed for each analysis. The full-length gels are presented in Supplementary Figure 2F

Next, we compared the relative mRNA and protein levels of USP18 between normal human cervical epithelial (HcerEpic) and cervical cancer cells, including Hela, C-33A, Caski, and SiHa. Both the relative mRNA and protein levels of USP18 were upregulated significantly in cervical cancer cells (except for Hela), especially Caski and SiHa cells (Fig. 2a & b). Therefore, USP18 expression increased in Caski and SiHa cells.

For silencing, three siRNAs targeting different regions of the human *USP18* gene (siUSP18–1, siUSP18–1, and siUSP18–3) and siNC were synthesised and transfected into Caski and SiHa cells. Untreated cells functioned as a blank control (Blank). Both the relative mRNA and protein levels of USP18 were reduced significantly in Caski and SiHa cells transfected with siUSP18–1, siUSP18–2, and siUSP18–3 (Fig. 2c & d). Also, HcerEpic cells were transfected with an overexpressing USP18 plasmid (oeUSP18). A mock plasmid functioned as the negative control (oeNC). As shown in Fig. 2e & f, oeUSP18 remarkably promoted the ectopic expression of USP18 in HcerEpic cells.

### USP18 silencing inhibited proliferation and promoted apoptosis in human cervical cancer cells

**Fig. 3:**
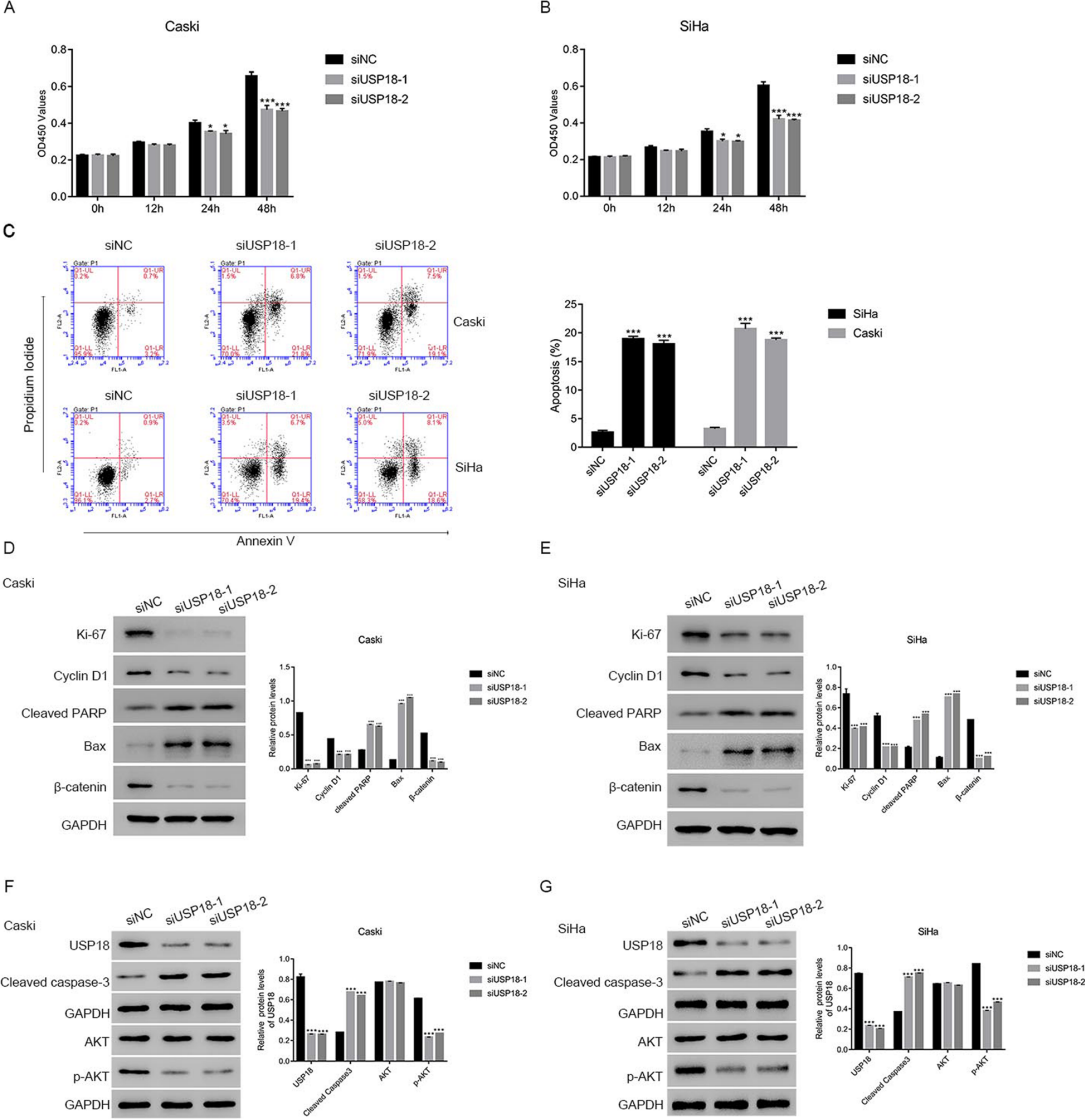
USP18 silencing suppressed the proliferation and induced apoptosis in human cervical cancer cells. **a** & **b** siUSP18–1 or siUSP18–2 significantly suppressed the proliferation of Caski and SiHa cells, *n* = 3 for each group. * *p* < 0.05 vs siNC; *** *p* < 0.001 vs siNC. **c** The apoptosis of Caski and SiHa cells were upregulated after transfecting with siUSP18–1 or siUSP18–2, *n* = 3 for each group. *** *p* < 0.001 vs siNC. **d** & **e** Western blot was used to examine the protein contents of Ki-67, Cyclin D1, cleaved PARP, BAX and β-catenin in Caski and SiHa cells that transfected with siUSP18–1 or siUSP18–2 respectively, *n* = 3 for each group. *** *p* < 0.001 vs siNC. The full-length gels are presented in Supplementary Figure 3D-E. **f** & **g** Western blot was used to examine the protein contents of USP18, Cleaved caspase-3, AKT and p-AKT in Caski and SiHa cells that transfected with siUSP18–1 or siUSP18–2 respectively, *n* = 3 for each group. *** *p* < 0.001 vs siNC. The full-length gels are presented in Supplementary Figure 3F-G

Then, we examined the proliferation rate of Caski and SiHa cells transfected with siUSP18–1 or siUSP18–2, using CCK-8 assays. As presented in Fig. 3a & b, both siUSP18–1 and siUSP18–2 significantly abolished the proliferation of Caski and SiHa cells. Moreover, the apoptosis of cells transfected with siUSP18–1 and siUSP18–2 was substantially higher than that in siNC-transfected Caski and SiHa cells (Fig. 3c).

Ki-67 and Cyclin D1 are well-known cell proliferation markers [13]. As presented in Fig. 3d, the proteins levels of Ki-67 and Cyclin D1 decreased significantly in Caski cells transfected with siUSP18–1 or siUSP18–2. Moreover, we also measured the levels of pro- or anti-apoptotic biomarkers in Caski cells, as indicated. Both siUSP18–1 and siUSP18–2 increased the expression of the pro-apoptosis factors cleaved PARP and Bax. Interestingly, the protein level of β-catenin, an AKT downstream factor, was also significantly decreased in USP18 siRNA-transfected cells. Importantly, similar results were obtained in SiHa cells (Fig. 3e). These results suggested that USP18 was a pro-proliferation and anti-apoptosis mediator in human cervical cancer cells.

Cleaved caspase-3 is a positive apoptotic factor [14]. In the current study, Western blotting was performed to quantify the protein contents of USP10, cleaved caspase-3, p-AKT and AKT in Caski and SiHa cells transfected with siUSP18–1 or siUSP18–2. Our results suggested that the protein content of cleaved caspase-3 increased significantly in siUSP18–1- or siUSP18–2-transfected cells. Interestingly, USP18 siRNAs suppressed the phosphorylation of AKT in Caski and SiHa cells (Fig. 3f & g).

To further confirm the function of USP18 in Caski cells, a rescue assay was established by transfecting oeUSP18 into USP18-silenced Caski cells. Our results indicated that oeUSP18 rescued the function of USP18 in siUSP18-transfected cells, further demonstrating USP18’s role as an oncogene in human cervical cancer cells (Figure S1).

### The PI3/AKT inhibitor LY294002 disrupted the function of oeUSP18 in HcerEpic cells

**Fig. 4:**
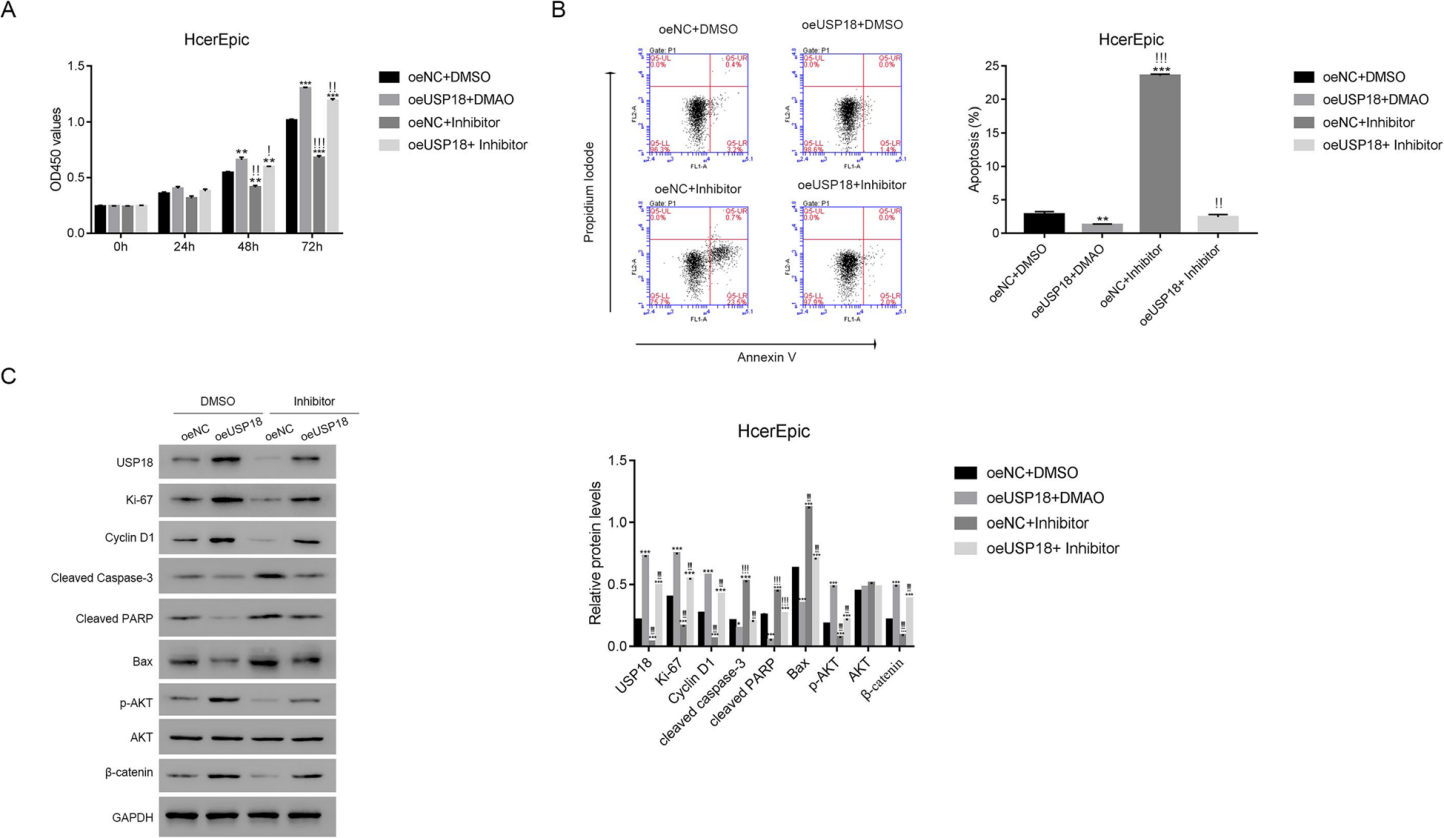
The PI3K/AKT inhibitor LY294002 disrupted the function of oeUSP18 in human HcerEpic cells. **a** The proliferation of oeUSP18 transfected cells was suppressed in the presence of the PI3K/AKT inhibitor LY294002. * *p* < 0.05 vs oeNC + DMSO, ** *p* < 0.01 vs oeNC + DMSO, *** *p* < 0.001 vs oeNC + DMSO; ### *p* < 0.001 vs oeUSP18 + DMSO. *N* = 3 for each time point **b** The PI3K/AKT inhibitor LY294002 promoted the apoptosis of oeUSP14 transfected cells. * *p* < 0.05 vs oeNC + DMSO, *** *p* < 0.001 vs oeNC + DMSO; ### *p* < 0.001 vs oeUSP18 + DMSO. *N* = 3 for each group. **c** Western blot was used to examine the protein contents of USP18, Ki-67, Cyclin D1, cleaved caspase-3, cleaved PARP, Bax, p-AKT, AKT and β-catenin in oeNC or oeUSP18 transfected cells with or without the treatment of the PI3K/AKT inhibitor LY294002. *** *p* < 0.001 vs oeNC + DMSO; ### *p* < 0.001 vs oeUSP18 + DMSO. *N* = 3 for each group. The full-length gels are presented in Supplementary Figure 4C

The PI3/AKT inhibitor LY294002 was used to block AKT’s endogenous activity to assess the relationship between USP18 and AKT in cervical cancer cells. As shown in Fig. 4a, USP18 overexpression improved HcerEpic cell proliferation, but the inhibitor LY294002 disrupted this function. Moreover, oeUSP18 significantly reduced the apoptosis of HcerEpic cells, whereas LY294002 reversed this suppression (Fig. 4b).

Moreover, oeUSP18 promoted the protein levels of Ki-67 and Cyclin D1, while inhibited the levels of cleaved PARP and Bax. Further, the protein level of cleaved caspase-3 was suppressed in HcerEpic cells transfected with oeUSP18. However, LY294002 remarkably significantly disrupted oeUSP18 fucntion in HcerEpic cells. Importantly, AKT phosphorylation was higher in oeUSP18-transfected cells but significantly suppressed by LY294002 (Fig. 4c).

### Silencing of USP18 inhibited the tumorigenicity of human cervical cancer cells in vivo

**Fig. 5:**
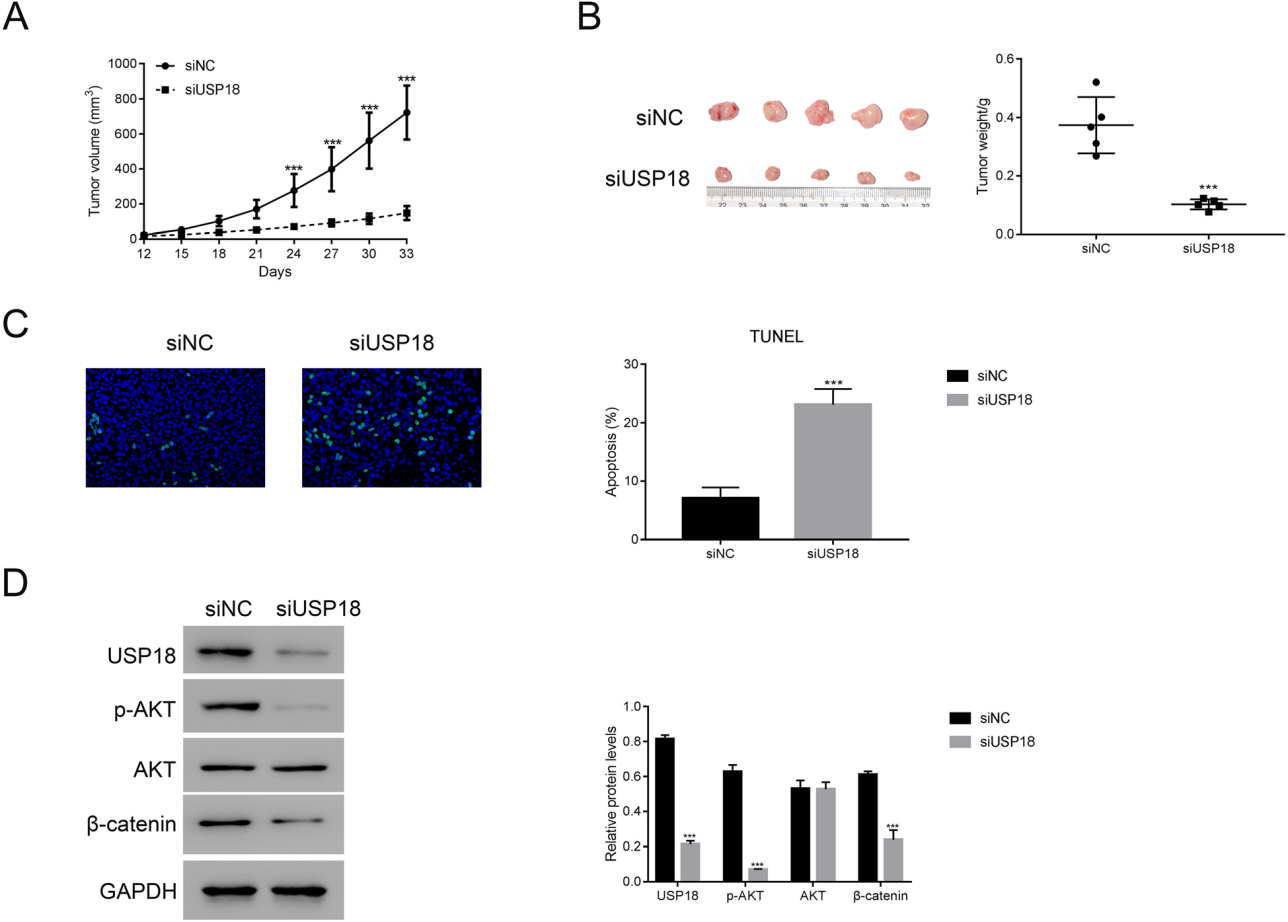
Silencing of USP18 inhibited tumorigenicity of human cervical cancer cells in vivo. **a** & **b** the tumor volume and weight in nude mice injected with Caski cells transfected with siNC and siUSP18. *** *p* < 0.001 vs siNC. *N* = 5 for each group. **c** TUNEL staining assay was performed to examine the apoptosis of siNC and siUSP18 tumours. *** *p* < 0.001 vs siNC. Three replications were analysed for each analysis. **d** Western blot was used to examine the protein levels of USP18, p-AKT, AKT, β-catenin in siNC and siUSP18 tumours. *** *p* < 0.001 vs siNC. Three replications were analysed for each analysis. The full-length gels are presented in Supplementary Figure 5D

An equal number of Caski cells, transfected with siNC or siUSP18, was hypodermically injected into nude mice (*n* = 5 for each group) to investigate USP18’s role in regulating tumorigenicity in vivo*.* Tumour formation was assessed every 3 days for 33 days (started on day 12). Although both the injected cell types were capable of developing tumours, it was evident that USP18-siRNA cells suppressed the tumour growth rate (Fig. 5a & b). The volume and weight of USP18-siRNA tumours were reduced significantly compared with those of siNC tumours. Moreover, the TUNEL staining assay results indicated that the apoptosis of cells in siUSP18 tumours was substantially higher than that in siNC tumours (Fig. 5c).

Further, the protein content of USP18 was downregulated in siUSP18 tumours. Additionally, the phosphorylation levels of AKT and β-catenin also decreased in siUSP18 tumours (Fig. 5d). Together, these results suggested that USP18 silencing inhibited the tumorigenicity of human cervical cancer cells in vivo*.*

## Discussion

Cervical cancer is a common malignant tumour in women with a high mortality rate worldwide. Currently, the main treatments for cervical cancer include surgery, postoperative radiotherapy, and chemotherapy. However, these therapies are associated with damaging side effects and cause significant pain for patients [15]. Therefore, novel therapies are needed urgently.

The ubiquitin-proteasome system is critical for regulating tumour cells’ biological processes [16]. Dysregulated ubiquitination and de-ubiquitination are closely associated with cervical cancer progression [17, 18]. In the present study, USP18 was suggested as an oncogene in human cervical cancer. Therefore, targeting USP18 provided novel insight into the treatment of cervical cancer.

A previous report demonstrated that silencing USP18 suppressed proliferation and promoted apoptosis in hepatocellular cancer cells [19]. In the current study, the downregulation of USP18 also significantly suppressed the proliferation of cervical cancer cells and induced cell apoptosis. Moreover, USP18 overexpression presented the opposite effects. Therefore, these results demonstrated that USP18 was a pro-proliferation and anti-apoptosis factor in cervical cancer cells.

Growing evidence has indicated that the PI3K/AKT pathway plays a crucial function in tumour progression and metastasis [20, 21]. Targeting PI3K/AKT signalling has been confirmed as a potential therapeutic strategy for multiple human cancers, including pancreatic cancer, breast cancer, and bladder cancer [22–24]. Moreover, activated PI3K/AKT correlates with the progression and metastasis of cervical cancer cells [25]. In this study, USP18 knockdown significantly suppressed theAKT phosphorylation in cervical cancer cells. Importantly, the PI3K/AKT inhibitor LY294002 significantly suppressed the function of oeUSP18 in cervical cancer cells. Hence, USP18 was involved in the regulation of the PI3K/AKT pathway in cervical cancer cells. Our results suggest that USP18 might promote cell proliferation and inhibit the apoptosis of cervical cancer cells by regulating the PI3K/AKT pathway.

## Conclusions

In the present study, we explored USP18’s function in human cervical cancer cells. First, our findings indicated that USP18 was increased in human cervical cancer tissues and promoted the progression of human cervical cancer cells. Second, our study not only elucidated the possible signalling pathway of USP18 in human cervical cancer cells but also provided evidence of its potential use as a target in cervical cancer treatment.

## Supplementary information

**Additional file 1: Figure S1.** oeUSP18 rescused the function of USP18 siUSP18 transfected cells. A. CCK-8 was used to determine the proliferation of Caski cells that transfected with siNC, siUSP18–2, and siUSP18 + oeUSP18. * *p* < 0.05 vs siNC, *** *p* < 0.001 vs siNC;!!! *p* < 0.001 vs siUSP18–2. Three replications were analysed for each time point. B. Flow cytometer was used to examine the apoptosis of Caski cells that transfected with siNC, siUSP18–2, and siUSP18 + oeUSP18, respectively. Three replications were analysed for each analysis C. Western blot was used to examine the protein contents of USP18, cleaved caspase-3, p-AKT and AKT in Caski cells that transfected with siNC, siUSP18–2 and siUSP18 + oeUSP18 respectively, *** *p* < 0.001 vs. siNC. Three replications were analysed for each analysis. The full-length gels are presented in Supplementary Figure S1-C.

**Additional file 2.**

## Data Availability

The datasets used and/or analysed during the current study are available from the corresponding author on reasonable request.

## Abbreviations

DUB: Deubiquitinating
USP18: Ubiquitin-specific protease 18
qRT-PCR: Quantitative real time polymerase chain reaction
RNAi: RNA interference
CCK-8: Cell Counting Kit-8
HPV: Human papillomavirus
cDNA: Complementary DNA
TCGA: The Cancer Genome Atlas
ES: Enrichment score
ANOVA: Analysis of variance
CESC: Cervical squamous cell carcinoma

## References

[1] Manzo-Merino J, Contreras-Paredes A, Vázquez-Ulloa E, Rocha-Zavaleta L, Fuentes-Gonzalez AM, Lizano M (2014). The role of signaling pathways in cervical cancer and molecular therapeutic targets. Arch Med Res.

[2] Khan SR, Rockall AG, Barwick TD (2016). Molecular imaging in cervical cancer. Q J Nucl Med Mol Imaging.

[3] Menderes G, Black J, Schwab CL, Santin AD (2016). Immunotherapy and targeted therapy for cervical cancer: an update. Expert Rev Anticancer Ther.

[4] Tokarz S, Berset C, La Rue J, Friedman K, Nakayama K-I, Nakayama K, Zhang D-E, Lanker S (2004). The ISG15 isopeptidase UBP43 is regulated by proteolysis via the SCFSkp2 ubiquitin ligase. J Biol Chem.

[5] Hoeller D, Hecker C-M, Dikic I (2006). Ubiquitin and ubiquitin-like proteins in cancer pathogenesis. Nat Rev Cancer.

[6] Burkart C, Arimoto K-I, Tang T, Cong X, Xiao N, Liu Y-C, Kotenko SV, Ellies LG, Zhang D-E (2013). Usp18 deficient mammary epithelial cells create an antitumour environment driven by hypersensitivity to IFN-λ and elevated secretion of Cxcl10. Embo Mol Med.

[7] Potu H, Sgorbissa A, Brancolini C (2010). Identification of USP18 as an important regulator of the susceptibility to IFN-alpha and drug-induced apoptosis. Cancer Res.

[8] Sgorbissa A, Tomasella A, Potu H, Manini I, Brancolini C (2011). Type I IFNs signaling and apoptosis resistance in glioblastoma cells. Apoptosis.

[9] Guo Y, Dolinko AV, Chinyengetere F, Stanton B, Bomberger JM, Demidenko E, Zhou D-C, Gallagher R, Ma T, Galimberti F (2010). Blockade of the ubiquitin protease UBP43 destabilizes transcription factor PML/RARα and inhibits the growth of acute promyelocytic leukemia. Cancer Res.

[10] Tan Y, Guanglin Z, Xianming W, Weicai C, Haidong G (2018). USP18 promotes breast cancer growth by upregulating EGFR and activating the AKT/Skp2 pathway. Int J Oncol.

[11] Livak KJ, Schmittgen TD (2001). Analysis of relative gene expression data using real-time quantitative PCR and the 2(−Delta Delta C(T)) method. Methods.

[12] Liu L, Xie D, Xie H, Huang W, Zhang J, Jin W, Jiang W, Xie D (2019). ARHGAP10 inhibits the proliferation and metastasis of CRC cells via blocking the activity of RhoA/AKT signaling pathway. OncoTargets Ther.

[13] Shevra CR, Ghosh A, Kumar M (2015). Cyclin D1 and Ki-67 expression in normal, hyperplastic and neoplastic endometrium. J Postgrad Med.

[14] Porter AG, Jänicke RU (1999). Emerging roles of caspase-3 in apoptosis. Cell Death Differ.

[15] Li H, Xiaohua W, Xi C (2016). Advances in diagnosis and treatment of metastatic cervical cancer. J Gynecol Oncol.

[16] Ciechanover A (1994). The ubiquitin-proteasome proteolytic pathway. Cell.

[17] Rolén U, Kobzeva V, Gasparjan N, Ovaa H, Winberg G, Kisseljov F, Masucci MG (2006). Activity profiling of deubiquitinating enzymes in cervical carcinoma biopsies and cell lines. Mol Carcinog.

[18] Sun Y (2006). E3 ubiquitin ligases as cancer targets and biomarkers. Neoplasia.

[19] Cai J, Liu T, Jiang X, Guo C, Liu A, Xiao X (2017). Downregulation of USP18 inhibits growth and induces apoptosis in hepatitis B virus-related hepatocellular carcinoma cells by suppressing BCL2L1. Exp Cell Res.

[20] Asati V, Mahapatra DK, Bharti SK (2016). PI3K/Akt/mTOR and Ras/Raf/MEK/ERK signaling pathways inhibitors as anticancer agents: structural and pharmacological perspectives. Eur J Med Chem.

[21] Miriam M, Maria Chiara DS, Laura B, Federico G, Emilio H (2014). PI3K/AKT signaling pathway and cancer: an updated review. Ann Med.

[22] Avan A, Hassanian SM, Ghayour-Mobarhan M, Ferns GA, Maftouh M, Shahidsales S, Hosseini M, Ebrahimi S (2017). Targeting the Akt/PI3K signaling pathway as a potential therapeutic strategy for the treatment of pancreatic cancer. Curr Med Chem.

[23] Costa RLB, Han HS, Gradishar WJ (2018). Targeting the PI3K/AKT/mTOR pathway in triple-negative breast cancer: a review. Breast Cancer Res Treat.

[24] Sathe A, Nawroth R (2018). Targeting the PI3K/AKT/mTOR pathway in bladder cancer. Methods Mol Biol.

[25] Jiang C, Xu R, Li X-X, Wang Y-Y, Liang W-Q, Zeng J-D, Zhang S-S, Xu X-Y, Yang Y, Zhang M-Y (2017). p53R2 overexpression in cervical cancer promotes AKT signaling and EMT, and is correlated with tumor progression, metastasis and poor prognosis. Cell Cycle.

