# Supplementary figures and images for: USP18 promotes cell proliferation and suppressed apoptosis in cervical cancer cells via activating AKT signaling pathway
Source: BMC Cancer. 2020 Aug 8;20:741. doi: 10.1186/s12885-020-07241-1 (PMC7414560; doi:10.1186/s12885-020-07241-1)

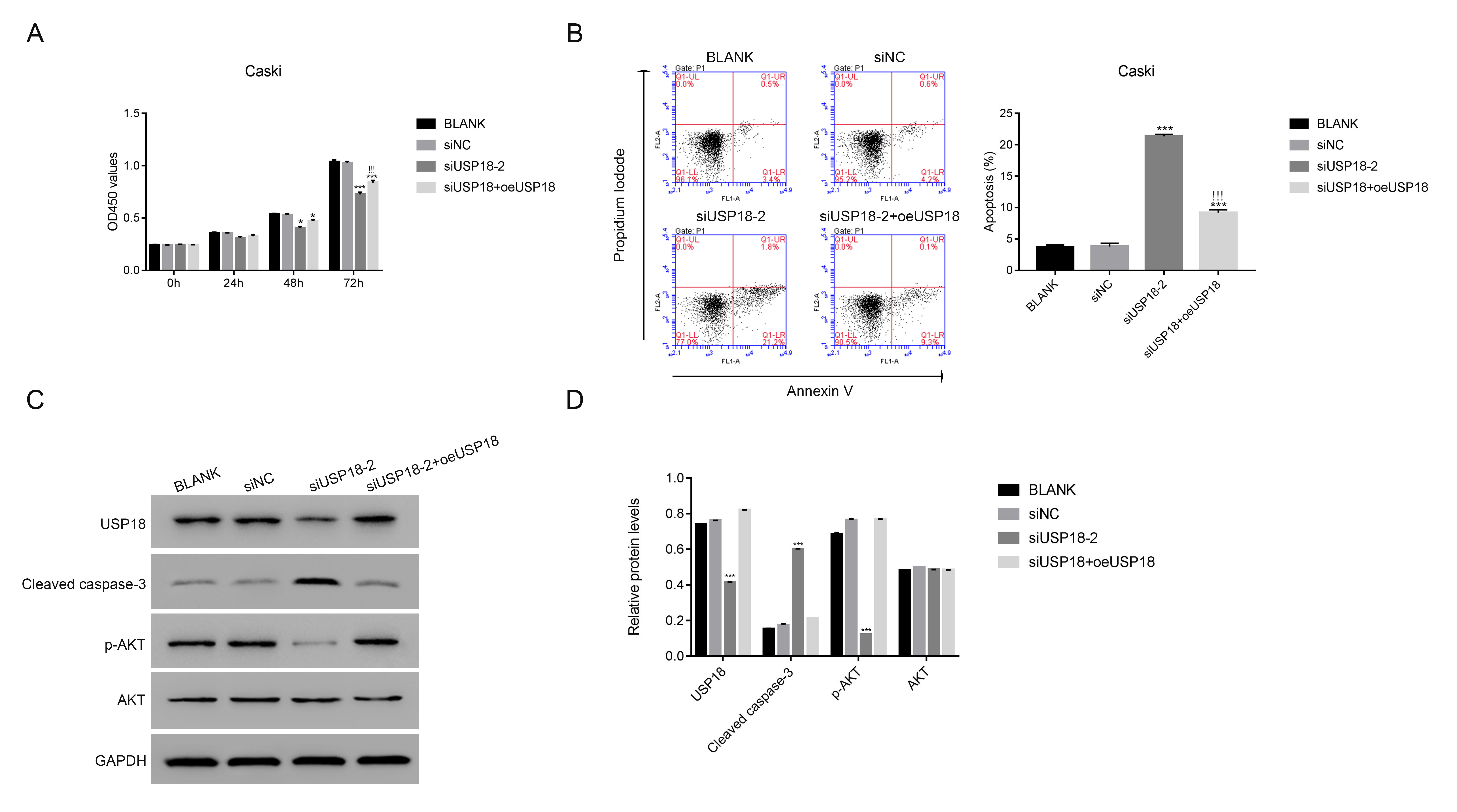

Supplement: Supplementary file 1 — Additional file 1: Figure S1. oeUSP18 rescused the function of USP18 siUSP18 transfected cells. A. CCK-8 was used to determine the proliferation of Caski cells that transfected with siNC, siUSP18–2, and siUSP18 + oeUSP18. * p < 0.05 vs siNC, *** p < 0.001 vs siNC;!!! p < 0.001 vs siUSP18–2. Three replications were analysed for each time point. B. Flow cytometer was used to examine the apoptosis of Caski cells that transfected with siNC, siUSP18–2, and siUSP18 + oeUSP18, respectively. Three replications were analysed for each analysis C. Western blot was used to examine the protein contents of USP18, cleaved caspase-3, p-AKT and AKT in Caski cells that transfected with siNC, siUSP18–2 and siUSP18 + oeUSP18 respectively, *** p < 0.001 vs. siNC. Three replications were analysed for each analysis. The full-length gels are presented in Supplementary Figure S1-C. [file 12885_2020_7241_MOESM1_ESM.jpg]
